# 2-(5-Methyl-1,3,4-oxa­diazol-2-yl)phenyl acetate

**DOI:** 10.1107/S1600536814007946

**Published:** 2014-04-16

**Authors:** Alexsandro F. dos Santos, Rodrigo Cristiano, Petrônio F. Athayde-Filho, Adailton J. Bortoluzzi

**Affiliations:** a Depto. de Química - Campus I - Universidade Federal da Paraíba, 58051-900 - João Pessoa, PB, Brazil; b Depto. de Química - Universidade Federal de Santa Catarina, 88040-900 - Florianópolis, Santa Catarina, Brazil

## Abstract

In the title compound, C_11_H_10_N_2_O_3_, which is a potential bioactive compound, the benzene and oxa­diazole rings are approximately coplanar, with an inter-ring dihedral angle of 4.14 (2)°, while the ester plane is rotated out of the benzene plane [dihedral angle = 82.69 (9)°]. In the crystal, the mol­ecules form layers down the *a* axis with weak π–π inter­actions between the oxa­diazole and benzene rings [minimum ring centroid separation = 3.7706 (14) Å].

## Related literature   

For the bioactivity of 1,3,4-oxa­diazole derivatives, see: Boström *et al.* (2012 ▶); Rajak *et al.* (2009 ▶); Polshettiwar & Varma (2008 ▶). For the properties of the 1,3,4-oxa­diazole heterocycle, see: Bolton & Kim (2007 ▶); Liu *et al.* (2007 ▶); Kulkarni *et al.* (2004 ▶). For material chemistry applications, see: Hughes & Bryce (2005 ▶); Wang *et al.* (2011 ▶); Cristiano *et al.* (2006 ▶); Han (2013 ▶). For the synthesis, see: Gallardo *et al.* (2001 ▶). For related structures, see: Vencato *et al.* (1996 ▶); Gutov (2013 ▶).
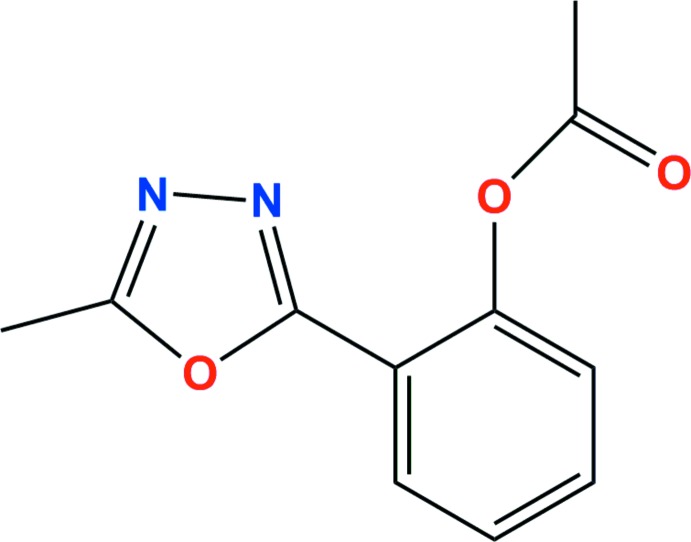


## Experimental   

### 

#### Crystal data  

C_11_H_10_N_2_O_3_
*M* *_r_* = 218.21
Monoclinic, 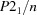

*a* = 6.6335 (6) Å
*b* = 16.925 (3) Å
*c* = 9.5078 (6) Å
β = 92.113 (6)°
*V* = 1066.7 (2) Å^3^
*Z* = 4
Mo *K*α radiation
μ = 0.10 mm^−1^
*T* = 293 K
0.50 × 0.36 × 0.16 mm

#### Data collection  

Enraf–Nonius CAD-4 diffractometer
1998 measured reflections
1885 independent reflections
1403 reflections with *I* > 2σ(*I*)
*R*_int_ = 0.018
3 standard reflections every 200 reflections intensity decay: 1%

#### Refinement  

*R*[*F* ^2^ > 2σ(*F* ^2^)] = 0.044
*wR*(*F* ^2^) = 0.136
*S* = 1.11
1885 reflections
146 parameters
H-atom parameters constrained
Δρ_max_ = 0.24 e Å^−3^
Δρ_min_ = −0.20 e Å^−3^

### 

Data collection: *CAD-4 Software* (Enraf–Nonius, 1989 ▶); cell refinement: *SET4* in *CAD-4 Software*; data reduction: *HELENA* (Spek, 1996 ▶); program(s) used to solve structure: *SIR97* (Altomare *et al.*, 1999 ▶); program(s) used to refine structure: *SHELXL97* (Sheldrick, 2008 ▶); molecular graphics: *PLATON* (Spek, 2009 ▶); software used to prepare material for publication: *SHELXL97*.

## Supplementary Material

Crystal structure: contains datablock(s) global, I. DOI: 10.1107/S1600536814007946/zs2285sup1.cif

Structure factors: contains datablock(s) I. DOI: 10.1107/S1600536814007946/zs2285Isup2.hkl

Supporting information file. DOI: 10.1107/S1600536814007946/zs2285Isup3.mol

Supporting information file. DOI: 10.1107/S1600536814007946/zs2285Isup4.cml

CCDC reference: 996388

Additional supporting information:  crystallographic information; 3D view; checkCIF report

## Figures and Tables

**Fig. 1.**
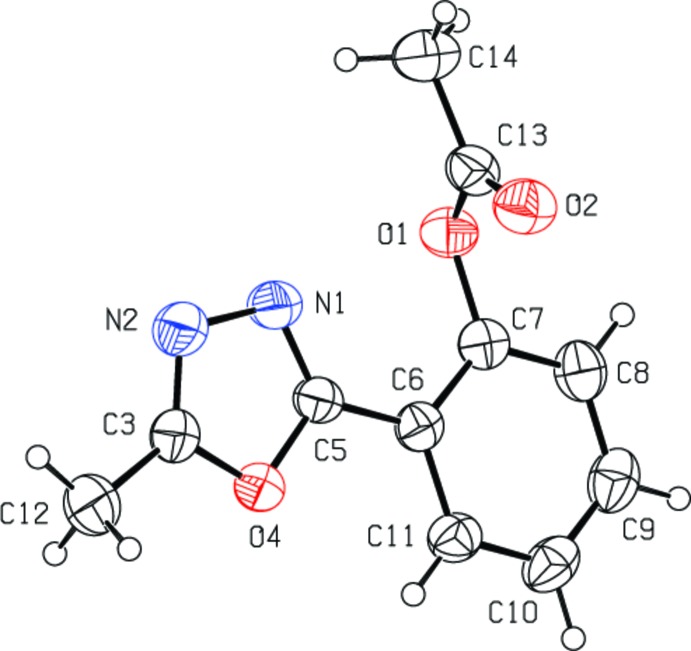
The molecular structure of the title compound with atom labeling scheme. Displacement ellipsoids are drawn at the 50% probability level.

## supplementary crystallographic information

### 1. Comment

Molecules containing the heterocycle 1,3,4-oxadiazole exhibit a wide range of biological activities, such as anticancer, antidiabetic, anti-inflammatory, analgesic, antibacterial, anticonvulsant, anti-HIV, herbicidal, fungicidal, pesticidal and antihypertensive (Boström *et al.*, 2012; Rajak *et al.*, 2009). This five-membered ring has been studied as a potential pharmacophore in a variety of chemical structures, due to its favorable metabolic profile and its capability of forming H-bonding associations (Polshettiwar & Varma, 2008; Gutov, 2013). Furthermore, aromatic substituted 1,3,4-oxadiazoles have widely been used in electro-optical devices due to their good thermal and chemical stability, blue luminescence with high quantum yield and electron transporting capabilities (Hughes & Bryce, 2005; Han, 2013).

As part of our continuing interest in the synthesis and evaluation of bioactive molecules containing *N*-heterocycles, we now report the synthesis and structure of the title compound C_11_H_10_N_2_O_3_. In this structure (Fig. 1), the benzene and oxadiazole rings are approximately coplanar, with an inter-ring dihedral angle of 4.14 (2)°, while the ester plane defined by O1, O2, C13, C14 is rotated out of the benzene plane giving a dihedral angle of 82.69 (9)° which corresponds to a torsion angle C6—C7—O1—C13 of 83.26 (22)°. In the crystal the molecules form layers down the *a* axis with weak inter-layer π–π interactions between the oxadiazole and benzene rings [minimum ring centroid separation = 3.7706 (14) Å].

### 2. Experimental

A mixture of 5-(2-hydroxyphenyl)tetrazole (Gallardo *et al.*, 2001) (2.0 g, 12.3 mmol) and acetic anhydride (6.3 g, 61.5 mmol) was heated under reflux for 2 h. The reaction mixture was poured into water/ice, the precipitate was filtered, washed with cold water and dried under vacuum to give the title compound as a white solid (1.88 g, 70%). Crystals suitable for X-ray diffraction were obtained from slow evaporation of the CDCl_3_ solution. M.p.= 108 °C. ^1^H NMR (CDCl_3_) = 8.00 (dd, J = 7.8 and 1.6 Hz, 1H), 7.60 - 7.51 (m, 1H), 7.38 (t, J = 7.8 Hz, 1H), 7.23 (t, J = 7.8 Hz, 1H), 2.60 (s, 3H), 2.42 (s, 3H); ^13^C NMR (CDCl_3_) = 169.88, 163.35, 162.12, 148.68, 132.67, 129.21, 126.60, 124.22, 117.65, 21.20, 11.08.

### 3. Refinement

All non-H atoms were refined with anisotropic displacement parameters. Hydrogen atoms were placed at their idealized positions with distances of 0.93 Å for C—H_Ar_ and 0.96 Å for CH_3_ groups and allowed to ride. Their *U*_eq_ were fixed at 1.2 and 1.5 times *U*_iso_ of the preceding atom for aromatic and methyl groups, respectively. H atoms of the methyl groups were treated as ideally disordered over two sites.

### Crystal data

**Table:**

C_11_H_10_N_2_O_3_	*D*_x_ = 1.359 Mg m^−^^3^
*M**_r_* = 218.21	Melting point: 381 K
Monoclinic, *P*2_1_/*n*	Mo *K*α radiation, λ = 0.71069 Å
*a* = 6.6335 (6) Å	Cell parameters from 25 reflections
*b* = 16.925 (3) Å	θ = 6.5–15.6°
*c* = 9.5078 (6) Å	µ = 0.10 mm^−^^1^
β = 92.113 (6)°	*T* = 293 K
*V* = 1066.7 (2) Å^3^	Block, colorless
*Z* = 4	0.50 × 0.36 × 0.16 mm
*F*(000) = 456	

### Data collection

**Table:**

Enraf–Nonius CAD-4 diffractometer	θ_max_ = 25.1°, θ_min_ = 2.4°
Radiation source: fine-focus sealed tube	*h* = −7→7
ω–2θ scans	*k* = −20→0
1998 measured reflections	*l* = −11→0
1885 independent reflections	3 standard reflections every 200 reflections
1403 reflections with *I* > 2σ(*I*)	intensity decay: 1%
*R*_int_ = 0.018	

### Refinement

**Table:**

Refinement on *F*^2^	Hydrogen site location: inferred from neighbouring sites
Least-squares matrix: full	H-atom parameters constrained
*R*[*F*^2^ > 2σ(*F*^2^)] = 0.044	*w* = 1/[σ^2^(*F*_o_^2^) + (0.07*P*)^2^ + 0.1662*P*] where *P* = (*F*_o_^2^ + 2*F*_c_^2^)/3
*wR*(*F*^2^) = 0.136	(Δ/σ)_max_ < 0.001
*S* = 1.11	Δρ_max_ = 0.24 e Å^−^^3^
1885 reflections	Δρ_min_ = −0.20 e Å^−^^3^
146 parameters	Extinction correction: *SHELXL97* (Sheldrick, 2008), Fc^*^=kFc[1+0.001xFc^2^λ^3^/sin(2θ)]^-1/4^
0 restraints	Extinction coefficient: 0.020 (4)

### Fractional atomic coordinates and isotropic or equivalent isotropic displacement parameters (Å^2^)

**Table:**

	*x*	*y*	*z*	*U*_iso_*/*U*_eq_	Occ. (<1)
C3	0.7116 (3)	0.20207 (12)	0.5466 (2)	0.0529 (5)	
C5	0.7187 (3)	0.09558 (12)	0.42764 (19)	0.0449 (5)	
C6	0.7352 (3)	0.00994 (11)	0.4065 (2)	0.0438 (5)	
C7	0.7258 (3)	−0.02402 (12)	0.2733 (2)	0.0466 (5)	
C8	0.7460 (3)	−0.10475 (13)	0.2556 (2)	0.0572 (6)	
H8	0.7388	−0.1265	0.1658	0.069*	
C9	0.7766 (3)	−0.15261 (13)	0.3706 (3)	0.0613 (6)	
H9	0.7912	−0.2068	0.3587	0.074*	
C10	0.7858 (3)	−0.12053 (13)	0.5041 (3)	0.0602 (6)	
H10	0.8053	−0.1532	0.5820	0.072*	
C11	0.7660 (3)	−0.03993 (13)	0.5220 (2)	0.0509 (5)	
H11	0.7733	−0.0187	0.6123	0.061*	
C12	0.7142 (4)	0.25279 (14)	0.6729 (3)	0.0694 (7)	
H12A	0.7253	0.2204	0.7557	0.104*	0.5
H12B	0.5916	0.2829	0.6741	0.104*	0.5
H12C	0.8274	0.2881	0.6712	0.104*	0.5
H12D	0.7043	0.3072	0.6449	0.104*	0.5
H12E	0.8379	0.2447	0.7266	0.104*	0.5
H12F	0.6021	0.2395	0.7295	0.104*	0.5
C13	0.8374 (4)	0.06195 (13)	0.0975 (2)	0.0552 (6)	
C14	0.7638 (4)	0.11009 (17)	−0.0244 (3)	0.0776 (8)	
H14A	0.6205	0.1038	−0.0371	0.116*	0.5
H14B	0.8285	0.0929	−0.1077	0.116*	0.5
H14C	0.7949	0.1647	−0.0073	0.116*	0.5
H14D	0.8754	0.1371	−0.0643	0.116*	0.5
H14E	0.6674	0.1481	0.0063	0.116*	0.5
H14F	0.7011	0.0762	−0.0942	0.116*	0.5
N1	0.7036 (3)	0.15221 (10)	0.33808 (19)	0.0611 (5)	
N2	0.6990 (3)	0.22223 (11)	0.4170 (2)	0.0669 (6)	
O1	0.6816 (2)	0.02180 (8)	0.15409 (14)	0.0548 (4)	
O2	1.0070 (2)	0.05640 (10)	0.14221 (17)	0.0666 (5)	
O4	0.7239 (2)	0.12238 (8)	0.56256 (14)	0.0491 (4)	

### Atomic displacement parameters (Å^2^)

**Table:**

	*U*^11^	*U*^22^	*U*^33^	*U*^12^	*U*^13^	*U*^23^
C3	0.0587 (13)	0.0460 (12)	0.0541 (13)	0.0042 (10)	0.0026 (10)	−0.0013 (10)
C5	0.0451 (11)	0.0506 (11)	0.0391 (10)	0.0001 (8)	0.0021 (8)	0.0012 (9)
C6	0.0368 (10)	0.0470 (11)	0.0476 (11)	−0.0006 (8)	0.0021 (8)	0.0024 (9)
C7	0.0421 (10)	0.0490 (12)	0.0487 (12)	−0.0030 (8)	0.0011 (8)	0.0009 (9)
C8	0.0546 (12)	0.0531 (13)	0.0639 (14)	−0.0025 (10)	0.0026 (10)	−0.0103 (11)
C9	0.0547 (14)	0.0443 (12)	0.0853 (18)	0.0006 (10)	0.0070 (12)	0.0026 (12)
C10	0.0504 (12)	0.0543 (13)	0.0762 (16)	0.0013 (10)	0.0056 (11)	0.0214 (11)
C11	0.0452 (11)	0.0561 (13)	0.0517 (12)	−0.0011 (9)	0.0057 (9)	0.0100 (10)
C12	0.0863 (17)	0.0600 (14)	0.0617 (15)	0.0041 (13)	−0.0012 (12)	−0.0142 (11)
C13	0.0674 (15)	0.0549 (13)	0.0434 (11)	0.0016 (11)	0.0032 (10)	−0.0043 (9)
C14	0.0953 (19)	0.0802 (18)	0.0571 (14)	0.0043 (14)	0.0002 (13)	0.0150 (13)
N1	0.0915 (14)	0.0473 (10)	0.0446 (11)	0.0068 (9)	0.0045 (9)	0.0027 (8)
N2	0.0977 (15)	0.0453 (10)	0.0580 (12)	0.0072 (10)	0.0057 (10)	0.0011 (9)
O1	0.0577 (9)	0.0606 (9)	0.0456 (8)	−0.0032 (7)	−0.0046 (6)	0.0004 (7)
O2	0.0631 (11)	0.0777 (12)	0.0591 (10)	−0.0034 (8)	0.0038 (8)	0.0065 (8)
O4	0.0542 (8)	0.0504 (8)	0.0427 (8)	0.0029 (6)	0.0007 (6)	−0.0010 (6)

### Geometric parameters (Å, º)

**Table:**

C3—N2	1.278 (3)	C12—H12A	0.9600
C3—O4	1.359 (2)	C12—H12B	0.9600
C3—C12	1.476 (3)	C12—H12C	0.9600
C5—N1	1.283 (3)	C12—H12D	0.9600
C5—O4	1.360 (2)	C12—H12E	0.9600
C5—C6	1.468 (3)	C12—H12F	0.9600
C6—C7	1.390 (3)	C13—O2	1.191 (3)
C6—C11	1.395 (3)	C13—O1	1.364 (3)
C7—C8	1.384 (3)	C13—C14	1.485 (3)
C7—O1	1.395 (2)	C14—H14A	0.9600
C8—C9	1.370 (3)	C14—H14B	0.9600
C8—H8	0.9300	C14—H14C	0.9600
C9—C10	1.380 (3)	C14—H14D	0.9600
C9—H9	0.9300	C14—H14E	0.9600
C10—C11	1.382 (3)	C14—H14F	0.9600
C10—H10	0.9300	N1—N2	1.404 (3)
C11—H11	0.9300		
			
N2—C3—O4	111.94 (18)	H12C—C12—H12E	56.3
N2—C3—C12	128.9 (2)	H12D—C12—H12E	109.5
O4—C3—C12	119.19 (19)	C3—C12—H12F	109.5
N1—C5—O4	112.05 (17)	H12A—C12—H12F	56.3
N1—C5—C6	130.60 (18)	H12B—C12—H12F	56.3
O4—C5—C6	117.34 (16)	H12C—C12—H12F	141.1
C7—C6—C11	117.89 (19)	H12D—C12—H12F	109.5
C7—C6—C5	122.15 (17)	H12E—C12—H12F	109.5
C11—C6—C5	119.94 (18)	O2—C13—O1	122.6 (2)
C8—C7—C6	121.16 (19)	O2—C13—C14	126.9 (2)
C8—C7—O1	117.97 (18)	O1—C13—C14	110.4 (2)
C6—C7—O1	120.73 (17)	C13—C14—H14A	109.5
C9—C8—C7	120.0 (2)	C13—C14—H14B	109.5
C9—C8—H8	120.0	H14A—C14—H14B	109.5
C7—C8—H8	120.0	C13—C14—H14C	109.5
C8—C9—C10	120.1 (2)	H14A—C14—H14C	109.5
C8—C9—H9	119.9	H14B—C14—H14C	109.5
C10—C9—H9	119.9	C13—C14—H14D	109.5
C9—C10—C11	120.1 (2)	H14A—C14—H14D	141.1
C9—C10—H10	120.0	H14B—C14—H14D	56.3
C11—C10—H10	120.0	H14C—C14—H14D	56.3
C10—C11—C6	120.8 (2)	C13—C14—H14E	109.5
C10—C11—H11	119.6	H14A—C14—H14E	56.3
C6—C11—H11	119.6	H14B—C14—H14E	141.1
C3—C12—H12A	109.5	H14C—C14—H14E	56.3
C3—C12—H12B	109.5	H14D—C14—H14E	109.5
H12A—C12—H12B	109.5	C13—C14—H14F	109.5
C3—C12—H12C	109.5	H14A—C14—H14F	56.3
H12A—C12—H12C	109.5	H14B—C14—H14F	56.3
H12B—C12—H12C	109.5	H14C—C14—H14F	141.1
C3—C12—H12D	109.5	H14D—C14—H14F	109.5
H12A—C12—H12D	141.1	H14E—C14—H14F	109.5
H12B—C12—H12D	56.3	C5—N1—N2	106.16 (17)
H12C—C12—H12D	56.3	C3—N2—N1	106.76 (17)
C3—C12—H12E	109.5	C13—O1—C7	117.22 (16)
H12A—C12—H12E	56.3	C3—O4—C5	103.10 (15)
H12B—C12—H12E	141.1		
			
C7—O1—C13—C14	−177.68 (18)	N1—C5—C6—C11	175.0 (2)
C13—O1—C7—C6	83.3 (2)	O4—C5—C6—C11	−3.4 (3)
C13—O1—C7—C8	−101.1 (2)	N1—C5—C6—C7	−3.5 (3)
C7—O1—C13—O2	3.3 (3)	C11—C6—C7—O1	175.59 (17)
C3—O4—C5—C6	178.30 (17)	C7—C6—C11—C10	−0.2 (3)
C5—O4—C3—N2	0.4 (2)	C11—C6—C7—C8	0.1 (3)
C3—O4—C5—N1	−0.4 (2)	C5—C6—C7—C8	178.61 (19)
C5—O4—C3—C12	−179.60 (19)	C5—C6—C7—O1	−5.9 (3)
C5—N1—N2—C3	0.0 (2)	C5—C6—C11—C10	−178.75 (19)
N2—N1—C5—O4	0.2 (2)	C6—C7—C8—C9	−0.2 (3)
N2—N1—C5—C6	−178.2 (2)	O1—C7—C8—C9	−175.85 (18)
N1—N2—C3—O4	−0.2 (2)	C7—C8—C9—C10	0.5 (3)
N1—N2—C3—C12	179.7 (2)	C8—C9—C10—C11	−0.6 (3)
O4—C5—C6—C7	178.11 (18)	C9—C10—C11—C6	0.5 (3)
